# Automated mitral valve vortex ring extraction from 4D‐flow MRI

**DOI:** 10.1002/mrm.28361

**Published:** 2020-06-18

**Authors:** Corina Kräuter, Ursula Reiter, Clemens Reiter, Volha Nizhnikava, Marc Masana, Albrecht Schmidt, Michael Fuchsjäger, Rudolf Stollberger, Gert Reiter

**Affiliations:** ^1^ Institute of Medical Engineering Graz University of Technology Graz Austria; ^2^ Division of General Radiology Department of Radiology Medical University of Graz Graz Austria; ^3^ Computer Vision Center Universitat Autònoma de Barcelona Barcelona Spain; ^4^ Division of Cardiology Department of Internal Medicine Medical University of Graz Graz Austria; ^5^ Research & Development Siemens Healthcare Diagnostics Graz Austria

**Keywords:** 4D‐flow, blood flow, left ventricle, mitral valve vortex ring, Q criterion, vortex parameter

## Abstract

**Purpose:**

To present and validate a method for automated extraction and analysis of the temporal evolution of the mitral valve (MV) vortex ring from MR 4D‐flow data.

**Methods:**

The proposed algorithm uses the divergence‐free part of the velocity vector field for *Q* criterion‐based identification and tracking of MV vortex ring core and region within the left ventricle (LV). The 4D‐flow data of 20 subjects (10 healthy controls, 10 patients with ischemic heart disease) were used to validate the algorithm against visual analysis as well as to assess the method’s sensitivity to manual LV segmentation. Quantitative MV vortex ring parameters were analyzed with respect to both their differences between healthy subjects and patients and their correlation with transmitral peak velocities.

**Results:**

The algorithm successfully extracted MV vortex rings throughout the entire cardiac cycle, which agreed substantially with visual analysis (Cohen’s kappa = 0.77). Furthermore, vortex cores and regions were robustly detected even if a static end‐diastolic LV segmentation mask was applied to all frames (Dice coefficients 0.82 ± 0.08 and 0.94 ± 0.02 for core and region, respectively). Early diastolic MV vortex ring vorticity, kinetic energy and circularity index differed significantly between healthy controls and patients. In contrast to vortex shape parameters, vorticity and kinetic energy correlated strongly with transmitral peak velocities.

**Conclusion:**

An automated method for temporal MV vortex ring extraction demonstrating robustness with respect to LV segmentation strategies is introduced. Quantitative vortex parameter analysis indicates importance of the MV vortex ring for LV diastolic (dys)function.

## 1 INTRODUCTION

Blood flow from the left atrium into the left ventricle (LV) is accompanied by the formation of a 3D vortex ring at the tips of the mitral valve (MV) leaflets, which is assumed to support ventricular filling,^1^ to store kinetic energy^2^ and to help redirect mitral inflow toward the aorta.^3^, ^4^ As studies suggest that properties of the MV vortex ring may be altered in LV dysfunction,^5^, ^6^ development of methods for automated analysis of the MV vortex ring may not only enhance understanding of the MV vortex ring’s physiological functions, but also enable exploration of the diagnostic capabilities of its pathological alterations.

Time‐resolved three‐directional cardiovascular MR phase‐contrast imaging (4D‐flow) provides a unique tool to measure and visualize complex swirling blood flow patterns in vivo.^7^ Profound MV vortex ring analysis is, however, challenging due to the lack of methods for automated extraction of temporal MV vortex ring evolution. At present, qualitative MV vortex ring analysis is performed using 2D vector field representations^8^ and streamlines or pathlines for 3D vortical flow visualization.^9^, ^10^, ^11^ Moreover, existing quantitative approaches require manual annotation of vortex regions on a single 2D slice^9^, ^10^, ^11^ or involve semi‐automated vortex detection in 3D.^12^, ^13^

As there is no universal definition of a vortex,^14^, ^15^ even in the absence of noise and with infinite temporal and spatial resolution, different vortex criteria have been used to identify MV vortex rings.^12^, ^13^, ^16^ Vector pattern matching was used to extract the MV vortex ring in a single frame by setting a fixed threshold to the calculated similarity measure field.^16^ Töger et al^12^ computed Lagrangian coherent structures^17^ to identify MV vortex ring boundaries, which were delineated manually on 2D Lagrangian coherent structure representations. Elbaz et al^13^ used the *λ*_2_ criterion^18^ for MV vortex ring detection at peak early and late diastolic inflow, by manually adjusting the *λ*_2_ threshold for every subject. While an algorithm for automated *λ*_2_ thresholding in the segmented heart at peak diastolic inflow has been proposed,^19^ methods for automated extraction of the MV vortex ring throughout the cardiac cycle are lacking. Furthermore, all mentioned methods are region‐based and do not include extraction of the very center of the vortex, the vortex core skeleton (here denoted as “vortex core”), which would allow better observation of vortex deformation processes and distinction between MV vortex ring and other LV vortex structures.

We present a method for automated extraction of the temporal evolution of the MV vortex ring from 4D‐flow MRI data. The proposed algorithm uses the divergence‐free part of the velocity vector field for *Q* criterion‐based^20^ identification and tracking of MV vortex ring core and region within the LV. The aim of this study was to validate the automated MV vortex ring extraction algorithm against visual analysis, to test its robustness with respect to LV segmentation, and to analyze quantitative MV vortex ring parameters in healthy controls and patients with chronic ischemic heart disease (IHD).

## 2 METHODS

### 2.1 Study population

The 4D‐flow data of 10 healthy controls (3 males; age = 59 ± 7 years; heart rate = 69 ± 9 bpm) without history of cardiac disease and 10 patients with chronic IHD (7 males; age = 62 ± 8 years; heart rate = 60 ± 9 bpm) were included in the analysis (further details on the study population are given in the Supporting Information). The data were acquired in a prospective study (ClinicalTrials.gov identifiers NCT01728597 and NCT03253835), which was approved by the local ethical review board. All participants provided written informed consent.

### 2.2 4D‐flow MRI acquisition

All subjects underwent 4D‐flow imaging at 3 T (Magnetom Skyra; Siemens Healthcare, Erlangen, Germany) in the supine position and under free breathing, using an 18‐channel body array coil together with 12 elements of a 32‐channel spine coil. A stack of slices in three‐chamber‐view orientation covering the LV was acquired using a 2D retrospectively electrocardiographically‐gated phase‐contrast sequence with three‐directional simple four‐point velocity encoding.^21^ Velocity encoding was set to 100 cm/s in all directions and was adapted in the case of aliasing. Further protocol parameters were TR = 5.2 ms, TE = 3.1 ms, spatial resolution = 1.8 × 2.5 × 4 mm^3^, bandwidth = 605 Hz/pixel, GRAPPA with parallel acquisition factor of 2 and acquisition of external reference lines for sensitivity estimation, and temporal resolution = 41.8 ms interpolated to 30 cardiac frames. Two‐fold averaging was used to reduce breathing artifacts, resulting in an acquisition time of 44 ± 9 seconds per slice and 13.3 ± 3.4 minutes for the stack of slices covering the LV. Whereas the data of healthy controls were acquired without contrast‐medium administration, 0.2 mmol/kg of gadobutrol (Gadovist; Bayer Schering Pharma, Germany) was administered to patients with IHD 15 to 20 minutes before 4D‐flow imaging. Flip angles were set to 12° and 18º‐20° for healthy subjects and patients with IHD, respectively.

### 2.3 Data preparation and visual analysis

**Figure 1:**
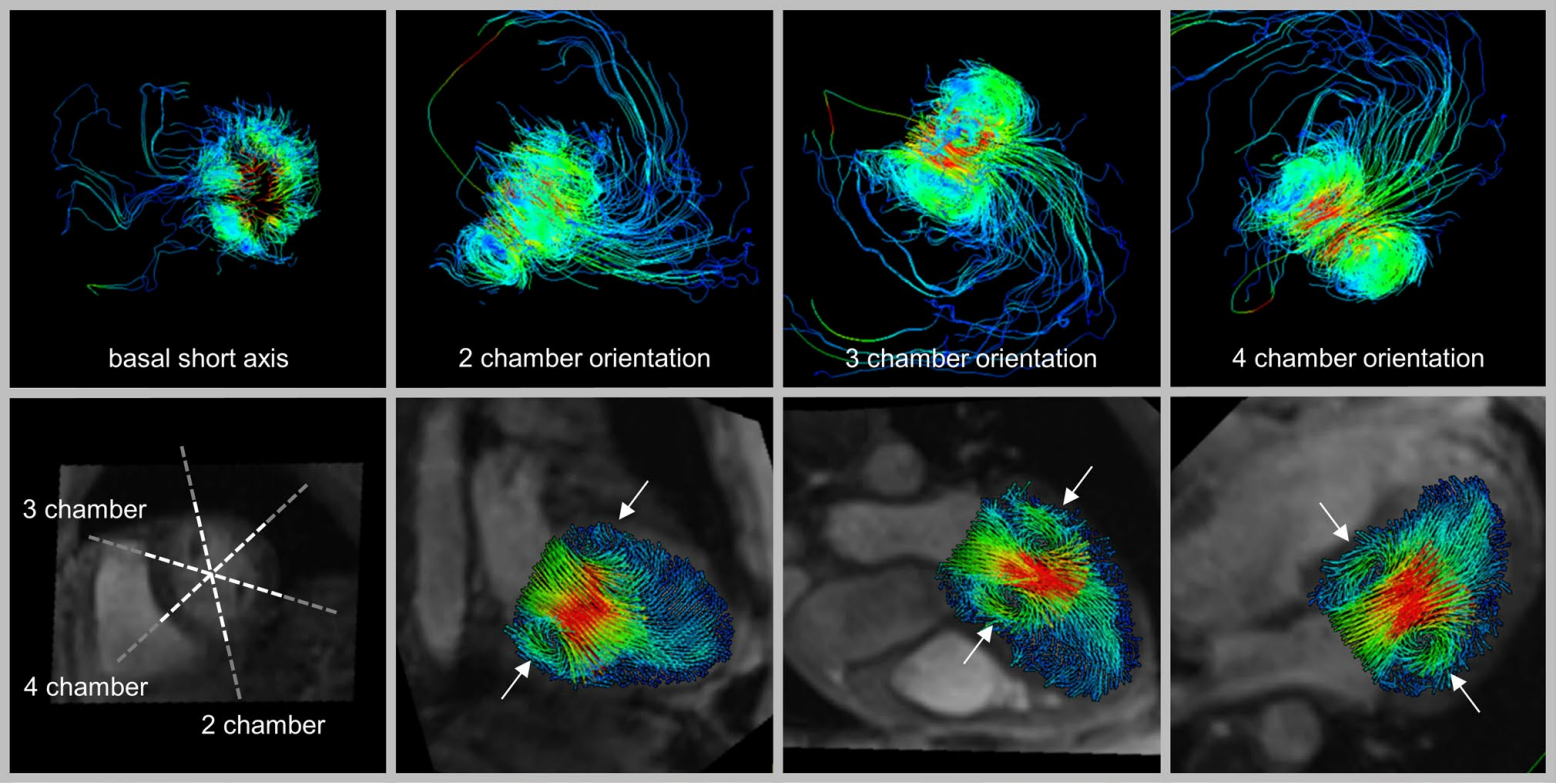
Visual analysis of the presence/absence of an MV vortex ring in one sample frame of a healthy subject. The 3D streamlines (upper panel) and multiplanar reconstructed 2D vector fields (lower panel) are color‐coded by velocity. An MV vortex ring was identified if swirling flow was present in at least four of six regions in the vicinity of the mitral valve (white arrows) in cardiac long‐axis views

Calculation and preprocessing (phase offset error correction, phase unwrapping) of the acquired velocity fields was performed by dedicated prototype software (*4DFlow*, Siemens Healthcare, Erlangen, Germany), which was further used for manual segmentation, measurement of transmitral flow parameters, as well as vector field and streamline visualization for visual analysis. The LV blood pool, including the LV outflow tract as well as papillary muscles and trabeculae, was manually segmented in all 30 cardiac frames on multiplanar reformatted short‐axis 4D‐flow magnitude images with a slice distance of *d* = 3.4 mm. Peak velocities of early and late diastolic transmitral inflow (E wave and A wave, respectively) were determined from the maximum velocity‐time curve measured at the MV leaflet tips and are denoted here by *v*_E_ and *v*_A_. Multiplanar reconstructed 2D velocity vector fields and 3D streamlines (seeded at the basal level of the LV) were visually evaluated in each frame for the presence of an MV vortex ring (Figure 1) by a reader with 15 years of experience in 4D‐flow MRI. By definition, a flow pattern was interpreted as an MV vortex ring if toroid flow was present in the vicinity of the MV leaflets.

All further data processing was automatically performed by in‐house software implemented in *MATLAB* (MathWorks, Natick, MA), based on the algorithm presented by Kräuter et al^22^, on a custom server with two Intel Xeon Silver 4216 (2 × 16 cores) and 24 × 16 GB of RAM.

### 2.4 Automated MV vortex ring extraction

**Figure 2:**
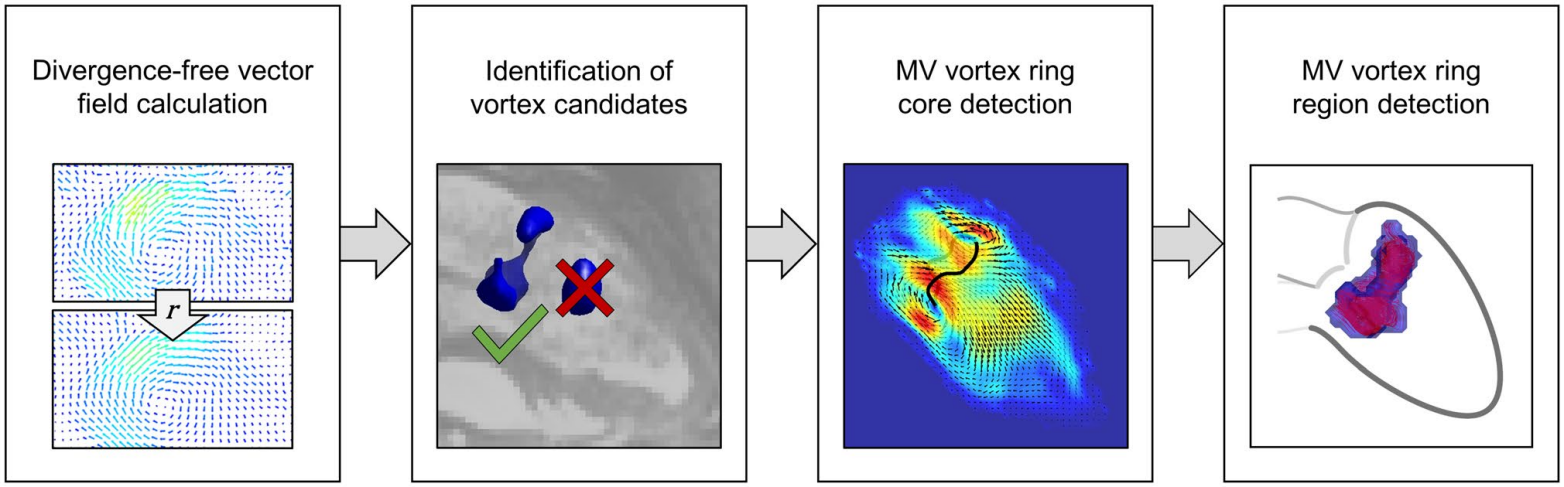
Pipeline of the proposed automated MV vortex ring extraction algorithm

The pipeline for automated MV vortex ring extraction consists of four steps (Figure 2). First, the divergence‐free part of the velocity vector field in a bounding box around the segmented LV is determined. Second, a scalar field describing vortex strength (*Q* criterion) is calculated and used to identify vortex candidates in the LV. Third, the MV vortex ring core is detected and traced over time. Finally, the MV vortex region is determined using streamlines seeded near the vortex core.

#### 2.4.1 Step 1: Divergence‐free vector field calculation

According to the Helmholtz‐Hodge decomposition,^23^ a smooth vector field v on a bounded or unbounded domain can be uniquely decomposed into a rotation‐free, a divergence‐free, and a harmonic part:

(1) v=d+r+h,

where d is rotation‐free ( ∇×d=0), r is divergence‐free ( ∇·r=0), and h is rotation‐free and divergence‐free ( ∇×h=0 and ∇·h=0). As we are aiming for vortex detection, only the part of the vector field containing information about rotation is of interest. To calculate the divergence‐free vector field, it can be represented as the curl of a vector potential

(2) r=∇×Ψ,

which leads to the Poisson equation

(3) ∇×∇×Ψ=∇×v.

With the boundary condition that the divergence‐free vector field must be tangential to the boundary, which is equal to ${\Psi |}_{\partial \Omega }=0$, the equation system can be uniquely solved.^24^ In our case, partial derivatives are approximated using finite differences, and the corresponding equation system is solved for Ψ using minimum norm least squares. The divergence‐free vector field is calculated from Ψ according to Equation (2).

#### 2.4.2 Step 2: Identification of vortex candidates

The *Q* value in each voxel inside the LV bounding box is calculated using the following equation:

(4) $$Q=\frac{1}{2}\left(\|\Omega^{2}\left\|-\right\|S^{2}\|\right),$$

where Ω is the spin tensor and S is the strain rate tensor of the divergence‐free vector field. According to the *Q* vortex criterion, voxels with *Q* > 0 are considered as part of a vortex.^20^ The obtained *Q* field is filtered in space with a Gaussian filter and a median filter, followed by temporal Gaussian smoothing, to remove noise and vortex structures with a short lifetime. After filtering, the LV segmentation masks are applied to the *Q* fields.

**Figure 3:**
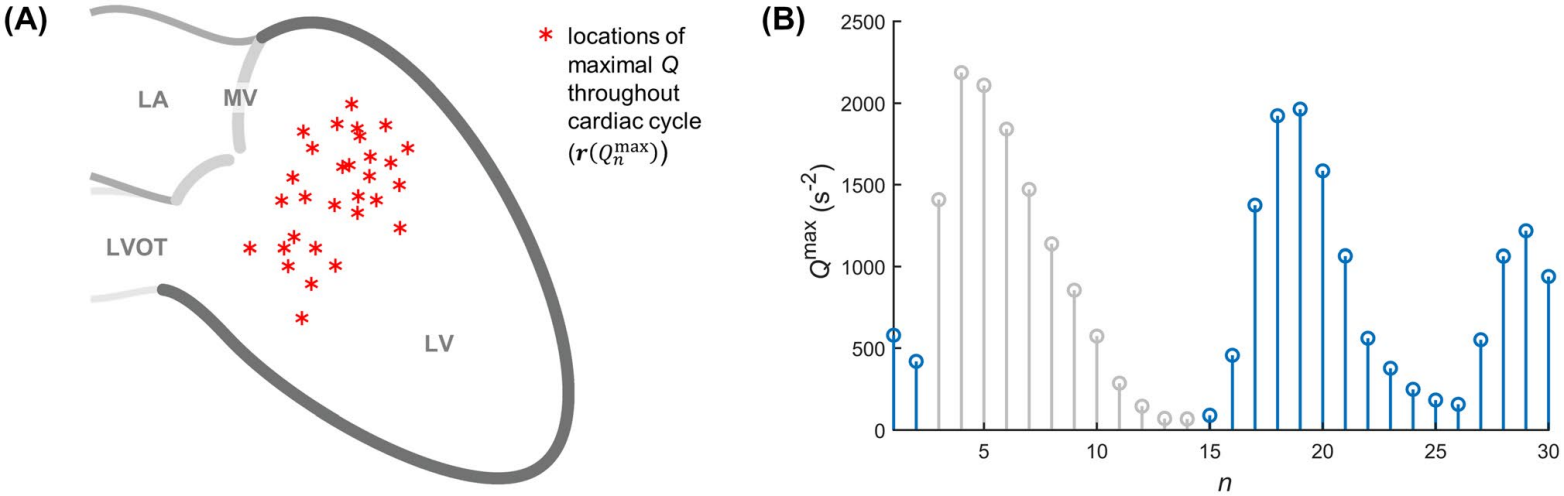
Maximum *Q* throughout the cardiac cycle for identification of vortex candidates. The locations of all *Q* maxima (A) allow calculation of a *Q* threshold in each frame that will reveal the strongest vortices in the LV. The maximum *Q* time‐curve (B) allows identification of diastolic to early systolic “vortex frames” (blue). Abbreviations: LA, left atrium; LVOT, left ventricular outflow tract

To identify regions of strong vortical flow, frame‐specific *Q* thresholds (*thr_Q,i_*) are determined according to

(5) $$thr_{Q,i}=\frac{1}{N}\sum_{n=1}^{N}Q_{i}\left(r\left(Q_{n}^{\max}\right)\right),$$

where *N* is the number of cardiac frames, *Q_i_* is the *Q* field in frame *i*, and $r\left(Q_{n}^{\max}\right)$ is the location of the maximum *Q* value in frame *n* (Figure 3). All further calculations are performed only on the early diastolic to early systolic “vortex frames,” which are identified from the curve of maximal *Q* values over time (Figure 3B).

As applying the frame‐specific thresholds to the *Q* fields typically yields several vortex regions, the next step is to identify “vortex candidates” that might belong to the MV vortex ring. This is done by performing principal component analysis of the thresholded *Q* fields over time, which represents the 4D information as a scalar 3D field for each principal component. In each frame, the field of the first principal component (*w*_1_) is used to calculate candidate factors *f*_c_, representing the probability that each vortex region belongs to the MV vortex ring as follows:

(6) $$f_{c}=\frac{1}{d_{\max}}\cdot \sum_{m=1}^{M}w_{1}\left(m\right),$$

where *d*_max_ is the maximal distance of the vortex region to the location of the maximum of the *w*_1_ field in LV long‐axis direction, and *M* is the number of vortex region voxels. The two vortex regions, if present, with highest probability of belonging to the MV vortex ring and a *d*_max_ that is smaller than two times the minimum *d*_max_ of all vortex regions, are selected as vortex candidates.

#### 2.4.3 Step 3: MV vortex ring core detection

**Figure 4:**
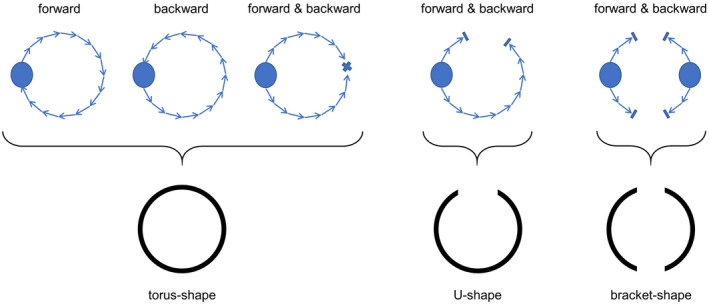
Possible MV vortex ring shapes defining the stop criteria of the predictor‐corrector algorithm. The predictor‐corrector method is applied to each vortex candidate independently, elongating the vortex core first in the forward and then in the backward direction. Stop criteria for forward elongation are arriving back at the start point, thereby forming a closed vortex ring, getting stuck or reaching a voxel where *Q* ≤ 0. In the latter two cases, the vortex core is elongated in the backward direction until a closed vortex ring is formed (by fusing with the forward core line or arriving at the start point) or the core line gets stuck or reaches a voxel with *Q* ≤ 0. If no closed core line is formed, the forward and backward core lines are merged and tested for having a U‐shape. If there are two vortex candidates and neither of them yields a torus‐shape or U‐shape, the option that together they form a bracket‐shape is assessed

A modified version of the predictor‐corrector method^25^ is applied for vortex core detection. In our implementation, the growing vortex core starts at the maximum *Q* point inside a vortex candidate region. In the predictor step, the vortex core is elongated in the direction of the local vorticity vector yielding the predictor point. In the corrector step, this point is corrected to the maximum *Q* point (= corrector point) on a plane that is perpendicular to the vorticity vector at the predictor point using a pattern‐search algorithm, as suggested by von Spiczak et al.^26^ The corrector point subsequently becomes the starting point for the next predictor‐corrector iteration. Possible shapes of an MV vortex ring core are defined as torus‐shape, U‐shape or bracket‐shape, and the predictor‐corrector algorithm uses stop criteria accordingly (Figure 4).

For robust MV vortex ring core detection, the predictor‐corrector algorithm is first applied to the two peak frames of the maximum *Q* time curve (Figure 3B). Then, vortex core detection is performed in each frame moving forward and backward in time, with the minimum between the peak frames marking the border between early diastolic forward and late diastolic backward iterations. If the current frame yields two distinct vortex cores not exhibiting a bracket‐shape, the vortex core whose centroid is closer to the centroid of the vortex ring core of the previous frame is defined as a possible MV vortex core.

The final step in each frame is to determine whether the detected vortex core belongs to the evolving MV vortex ring. As a measure of vortex deformation, the change of the largest principal axis length of an ellipsoid fitted to the vortex core from the previous frame to the current one is calculated. The vortex core of the current frame is considered to belong to the MV vortex ring if the previous frame yielded an MV vortex ring core and if the distance to the previous vortex core as well as the major axis length change are small.

#### 2.4.4 Step 4: MV vortex ring region detection

To identify the MV vortex ring region in each frame, streamlines are seeded on the divergence‐free vector field at the locations of MV vortex ring core voxels and their local neighborhood. Streamlines are extended until they reach a voxel with *Q* ≤ 0, identifying each voxel on their path as part of the MV vortex ring region. Finally, small vortex region branches that formed due to outlier streamlines are removed by iteratively taking out voxels with fewer than eight neighbors.

### 2.5 MV vortex ring parameters

The following parameters describing the MV vortex ring were automatically computed for each frame:

- *Maximum* and *mean vorticity* in the vortex region were determined from the vorticity magnitude in each MV vortex ring voxel. The corresponding vorticity field was calculated from the un‐decomposed velocity vector field using central finite differences. To account for outliers, the 95th percentile value was considered the maximum vorticity magnitude value.
- *MV vortex ring volume* (*vol*) was calculated as the sum of the vortex region voxels times the voxel volume.
- *Absolute* and *relative kinetic energy* (*E*_kin_) were computed from the velocity magnitude in each MV vortex ring voxel, assuming a density of blood of *ρ* = 1060 kg/m^3^.^27^ Relative *E*_kin_ was calculated by normalization to the MV vortex ring volume.
- *Angle to LV long axis* (α) was determined by fitting a plane to the MV vortex ring core and measuring the angle to the direction normal to the LV short‐axis plane.
- *Circularity index* (*CI*), as defined by Elbaz et al,^13^ was computed as the ratio between short and long axis of the MV vortex ring core. The long axis was determined as the largest principal axis length of an ellipsoid fitted to the vortex core, and the short axis as the second largest principal axis length.

### 2.6 Experiments on segmentation dependence

**Figure 5:**
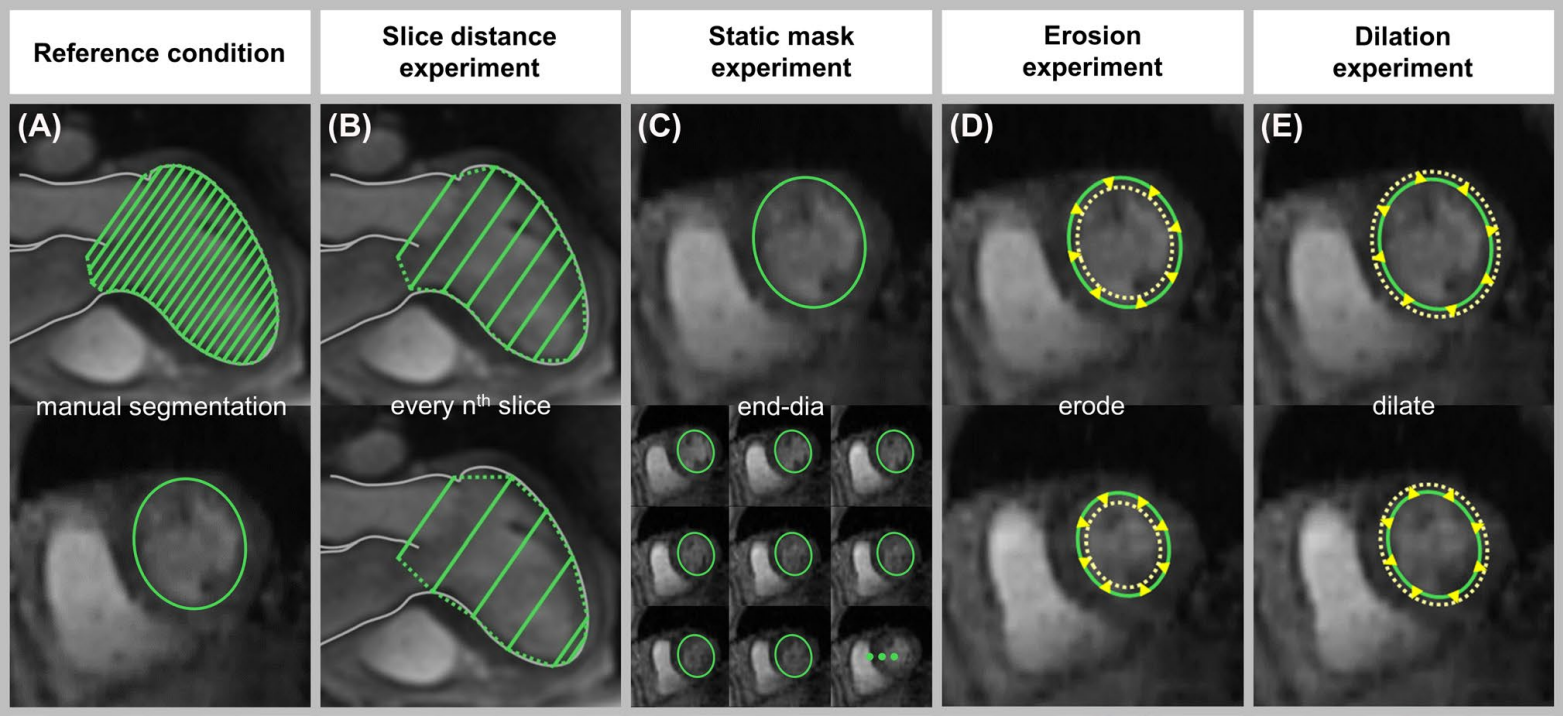
Reference condition and different segmentation approaches for MV vortex ring extraction and analysis experiments. In the reference condition (A), manual segmentation was performed on short‐axis images throughout the entire cardiac cycle. The slice distance was increased (B) and the segmentation mask at end‐diastole was applied to all cardiac frames (C) to simulate segmentation speed‐up. The segmentation masks of all frames were eroded (D) and dilated (E) to simulate segmentation variability

Several experiments using different segmentation masks were performed to assess the sensitivity of automated MV vortex ring extraction and analysis to LV segmentation, in which the manually segmented LV contours represented the reference condition (Figure 5A). A speed‐up of segmentation was simulated by stepwise increase of the slice distance *d* of short‐axis segmentation (Figure 5B), and by applying the fully segmented, static end‐diastolic mask (mask_end‐dia_) to all 30 cardiac frames (Figure 5C). In particular, the slice distance experiment (Figure 5B) was performed by taking each second (*2d*), third (*3d*), fourth (*4d*), fifth (*5d*), and sixth (*6d*) short‐axis slice, leading to a maximum slice distance of 20.4 mm. Furthermore, segmentation variability was simulated by erosion (mask_erode_) and dilation (mask_dilate_) of the reference segmentation mask by one voxel in each frame (Figure 5D,E).

### 2.7 Statistical analysis

Statistical analysis was performed with *MedCalc* (MedCalc Software, Ostend, Belgium) and *MATLAB*. The Cohen’s kappa coefficient (*κ*) was used for comparison of MV vortex ring existence in each frame between visual and automated analysis as well as for pairwise MV vortex ring existence comparisons of all segmentation masks with the reference condition. In MV vortex ring frames common to both segmentation masks, overlap ratios of vortex core and vortex region were assessed using the Dice similarity coefficient (*DSC*).^28^ Mean values of continuous variables resulting from visual and automated analysis, from application of different segmentation masks and from different cardiac frames, were compared by paired *t*‐test. Mean values of continuous variables were compared between healthy subjects and patients with IHD using unpaired *t*‐test. Dependencies of early and late diastolic MV vortex ring parameters on respective transmitral peak velocities and systolic LV function parameters were assessed by Pearson correlation coefficient (*r*). Pearson’s *r* was interpreted according to the following categorization: “poor” ≤ 0.5; 0.5 < “good” ≤ 0.7; 0.7 < “strong” ≤ 0.9; and “excellent” > 0.9. A *P* value < .05 was considered significant.

## 3 RESULTS

### 3.1 Automated MV vortex ring extraction and analysis

**Figure 6:**
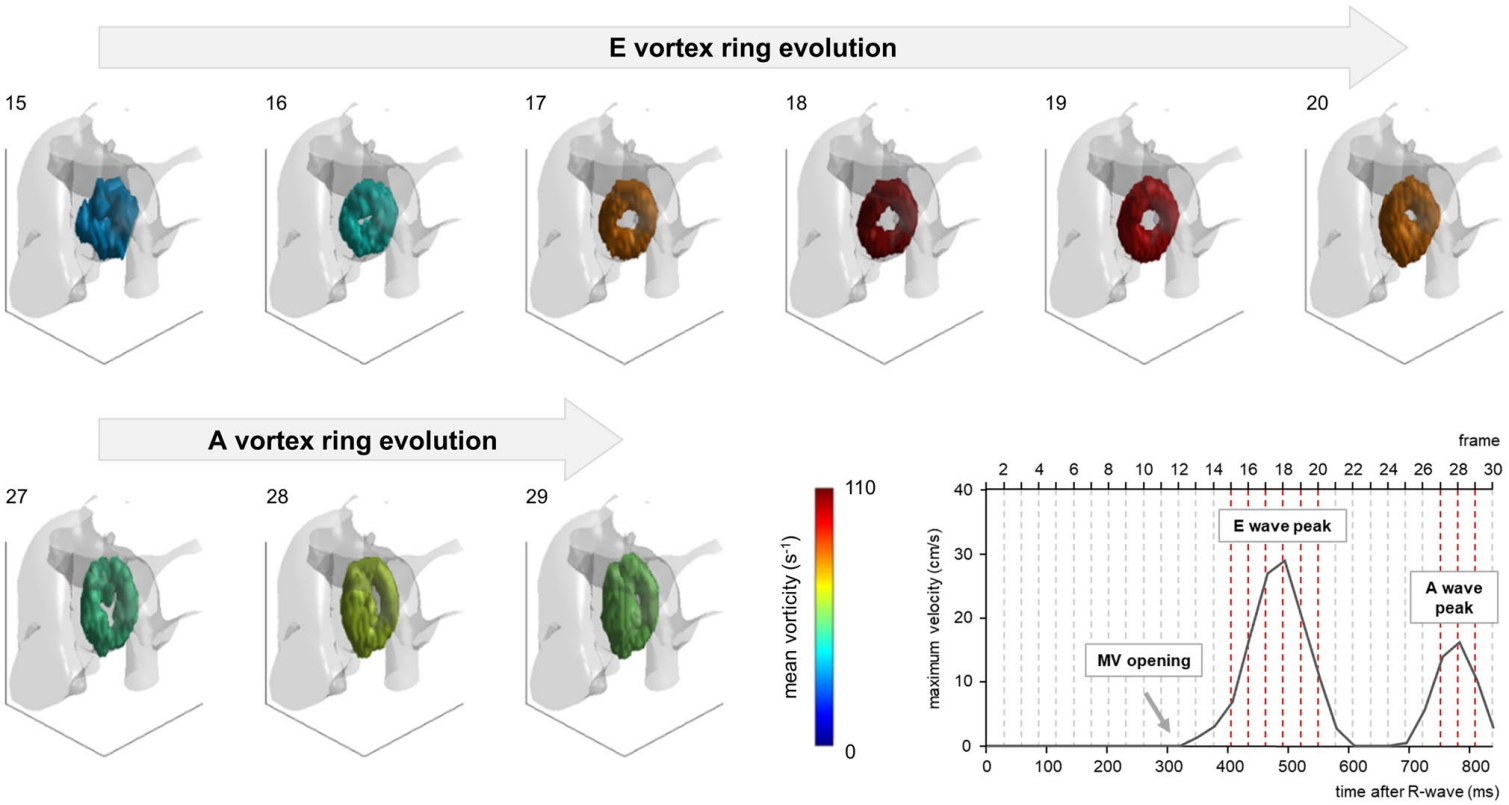
Temporal evolution of early and late diastolic MV vortex rings of a healthy subject. In each frame, the MV vortex ring is represented with *Q* isosurfaces that are color‐coded by MV vortex ring mean vorticity. For better orientation, the vortex rings are overlaid on a phase‐contrast MRA volume (notice the descending aorta on the right). The viewer position is at the apex looking upward. The diagram at the lower right shows the maximum forward velocity measured at the level of the mitral annulus

The proposed algorithm successfully extracted the temporal evolution of the MV vortex ring in healthy subjects and patients with IHD and determined vortex region–based as well as vortex core–derived parameters. Two periods of MV vortex ring existence were identified (Figure 6): the first starting during early diastole (E vortex ring), and the second starting during late diastole (A vortex ring), with a typical dissolution of the MV vortex ring during diastasis. Furthermore, MV vortex rings were present at both E and A wave peak velocity frames for all subjects. Velocity‐derived MV vortex ring parameters (vorticity and kinetic energy) also showed biphasic behavior with a peak during early diastole and another peak during late diastole, which did not necessarily coincide with respective E and A wave peak velocity frames (time difference up to 2 frames). In general, temporal evolution of vortex shape parameters (*vol*, α, and *CI*) did not exhibit biphasic behavior.

### 3.2 Comparison with visual analysis

Automated and visual analysis agreed substantially with respect to MV vortex ring existence in each frame (*κ* = 0.77 [0.72, 0.82], Table 1). In accordance with automated detection, visual analysis found E and A vortex rings and detected MV vortex rings at E and A wave peak velocity frames for all subjects. While onset times of MV vortex ring formation were found to be earlier by automated detection for both E and A vortex ring (E vortex ring bias = 30 ± 22 ms, *P* < .01; A vortex ring bias = 28 ± 14 ms, *P* < .01), MV vortex ring dissolution time differed between visual and automated detection only for E vortex ring (E vortex ring bias = −17 ± 34 ms, *P* = .04).

**Table 1:** The 2 × 2 confusion matrix of automatically and visually detected MV vortex rings in individual cardiac frames.

Automated	Visual		Total
	Absent	Present	
Absent	301	15	316
Present	54	230	284
Total	355	245	600

### 3.3 Dependence on segmentation

**Figure 7:**
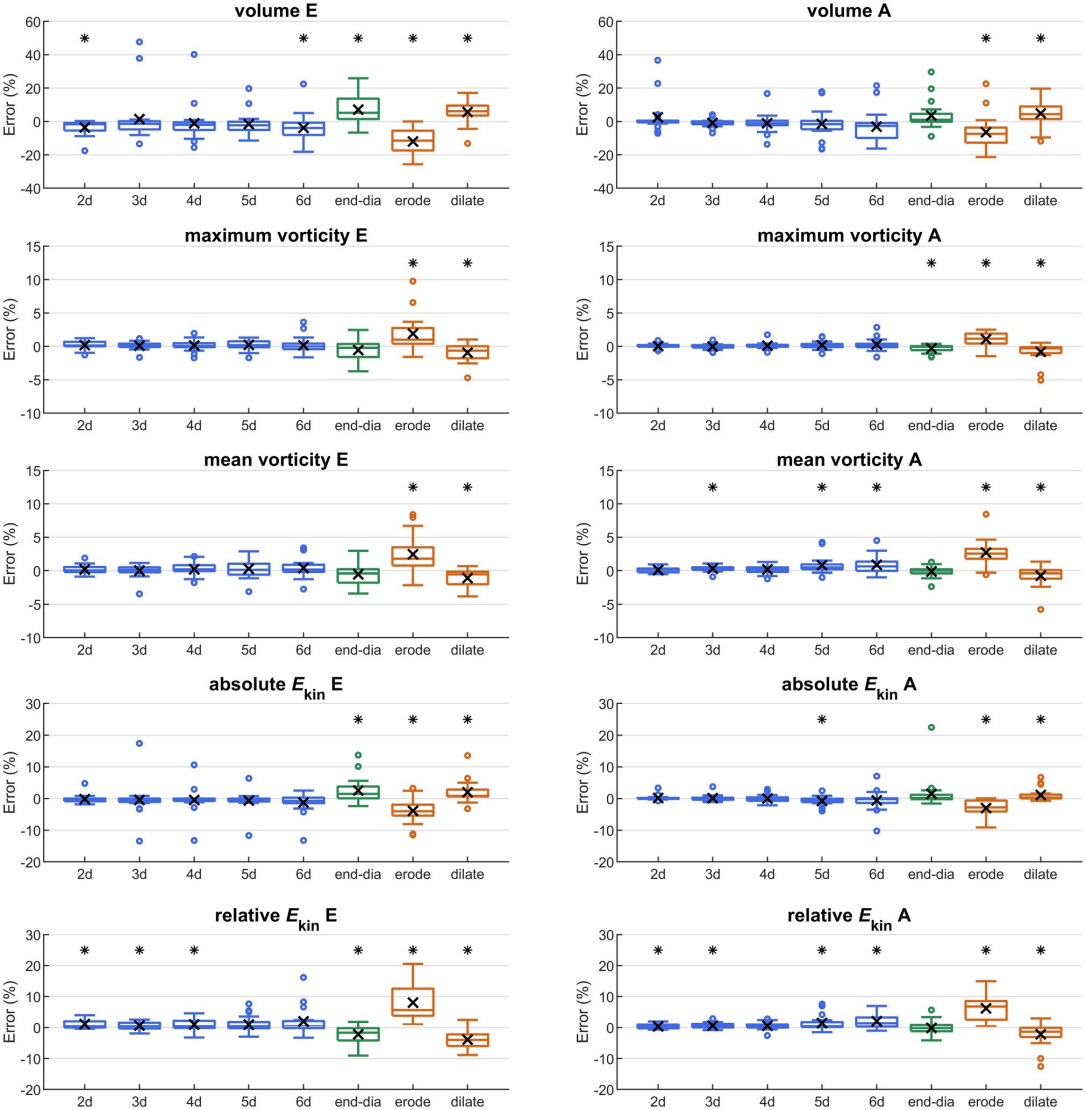
Error plots of early and late diastolic peak values of the MV vortex ring parameters volume, maximum vorticity, mean vorticity, absolute kinetic energy, and relative kinetic energy for different segmentation masks. Average errors are marked by ×, and a statistically significant difference to the reference condition is indicated by *

In the reference condition, 840 ± 118 short‐axis slices per subject were manually segmented. All segmentation experiments yielded E and A vortex rings in each subject. Table 2 provides comparisons with the reference condition regarding MV vortex ring existence and overlap of vortex core and region, as well as the estimated manual segmentation and processing times per subject. All segmentation masks yielded almost perfect agreement for MV vortex ring existence and, apart from mask_erode_, good to excellent MV vortex ring core and region overlap ratios. Increasing slice distance yielded a slight decrease in comparison scores. Whereas the processing time of the MV vortex ring extraction and analysis program is similar for all segmentation experiments, time for manual segmentation decreases drastically, using fewer slices or a static end‐diastolic mask. Figure 7 shows the error plots for early and late diastolic peak values of MV vortex ring parameters for all segmentation masks. For mask_erode_ and mask_dilate_, all MV vortex ring parameters were significantly different to the reference condition, with higher absolute mean errors (up to 12.0%) for mask_erode_. While the other segmentation masks also yielded sporadic statistically significant differences, their absolute mean errors were small (<7.2% for peak volume, <0.6% for peak maximum vorticity, <0.9% for peak mean vorticity, <2.6% for peak absolute kinetic energy, and <2.2% for peak relative kinetic energy). Comparing the average errors of different MV vortex ring parameters, maximum and mean vorticity were found to be the least sensitive to segmentation variation. Furthermore, all parameters were more stable for A vortex ring than for E vortex ring.

**Table 2:** Comparisons between the reference condition and each segmentation mask regarding MV vortex ring existence in each frame (*κ* values with their 95% confidence intervals), average spatial overlap indices of MV vortex ring core and MV vortex ring region, and estimated manual segmentation and processing times per subject.

Segmentation mask	Existence (*κ*)	Core (*DSC*)	Region (*DSC*)	Segmentation time (min)	Processing time (min)
reference	–	–	–	653.5 ± 92.1	71.5 ± 19.5
2d	0.96 [0.94, 0.99]	0.88 ± 0.06	0.97 ± 0.02	326.7 ± 46.1	71.4 ± 19.2
3d	0.96 [0.94, 0.98]	0.88 ± 0.05	0.97 ± 0.01	217.8 ± 30.7	71.5 ± 19.4
4d	0.94 [0.91, 0.97]	0.85 ± 0.07	0.96 ± 0.02	163.4 ± 23.0	71.3 ± 19.2
5d	0.91 [0.87, 0.94]	0.85 ± 0.07	0.96 ± 0.02	130.7 ± 18.4	71.4 ± 19.2
6d	0.90 [0.87, 0.94]	0.83 ± 0.08	0.95 ± 0.03	108.9 ± 15.4	71.5 ± 19.3
end‐dia	0.92 [0.89, 0.95]	0.82 ± 0.08	0.94 ± 0.02	21.8 ± 3.1	71.8 ± 19.1
erode	0.90 [0.87, 0.94]	0.74 ± 0.10	0.90 ± 0.03	653.5 ± 92.1	71.4 ± 19.5
dilate	0.90 [0.86, 0.94]	0.83 ± 0.09	0.94 ± 0.02	653.5 ± 92.1	72.0 ± 19.4

### 3.4 MV vortex ring parameters in healthy and diseased subjects

Early and late diastolic peak values of MV vortex ring parameters for healthy subjects and patients with IHD are given in Table 3. While A vortex ring parameters did not differ between healthy controls and patients, E vortex rings showed significant differences in MV vortex ring maximum vorticity, mean vorticity, absolute kinetic energy, relative kinetic energy, as well as circularity index.

**Table 3:** Early and late diastolic peak values of MV vortex ring parameters of healthy controls and patients with ischemic heart disease

Parameter	Controls (*N* = 10)	Patients with IHD (*N* = 10)	*P* value	*r* (*N* = 20)
E vortex ring volume (mL)	23 ± 3	23 ± 4	ns	ns
A vortex ring volume (mL)	20 ± 4	23 ± 4	ns	0.66 [0.31, 0.85]
E vortex ring max vorticity (s^−1^)	186 ± 30	135 ± 23	<.01	0.72 [0.40, 0.88]
A vortex ring max vorticity (s^−1^)	167 ± 36	163 ± 27	ns	0.76 [0.48, 0.90]
E vortex ring mean vorticity (s^−1^)	77 ± 11	60 ± 9	<.01	0.78 [0.52, 0.91]
A vortex ring mean vorticity (s^−1^)	73 ± 12	74 ± 10	ns	0.79 [0.54, 0.92]
E vortex ring absolute *E*_kin_ (mJ)	1.48 ± 0.80	0.87 ± 0.31	.04	0.68 [0.34, 0.86]
A vortex ring absolute *E*_kin_ (mJ)	1.01 ± 0.69	1.16 ± 0.37	ns	0.88 [0.72, 0.95]
E vortex ring relative *E*_kin_ (J/m^3^)	67 ± 25	42 ± 14	.01	0.78 [0.51, 0.91]
A vortex ring relative *E*_kin_ (J/m^3^)	53 ± 24	56 ± 18	ns	0.86 [0.68, 0.94]
E vortex ring α (°)	82 ± 4	81 ± 4	ns	ns
A vortex ring α (°)	81 ± 6	80 ± 5	ns	ns
E vortex ring *CI*	0.86 ± 0.04	0.78 ± 0.08	<.01	0.52 [0.10, 0.78]
A vortex ring *CI*	0.75 ± 0.06	0.71 ± 0.04	ns	ns

Note

The *P* value refers to unpaired *t*‐test, and *r* is the Pearson correlation coefficient (specified with its 95% confidence interval) for correlations between peak values of MV vortex ring parameters and the corresponding transmitral peak velocities.

Abbreviations: *CI*, circularity index; IHD, ischemic heart disease; ns, not statistically significant.

Both E and A wave peak velocities did not differ significantly between healthy and IHD subjects (*v*_E,healthy_ = 67 ± 7 cm/s, *v*_E,IHD_ = 58 ± 12 cm/s, *P* = .07; *v*_A,healthy_ = 61 ± 15 cm/s, *v*_A,IHD_ = 67 ± 11 cm/s, *P* = .28); however, the E‐to‐A‐wave peak velocity ratio differed (1.15 ± 0.25 vs. 0.89 ± 0.26, *P* = .03). In contrast to peak values of MV vortex ring shape parameters, peak values of velocity‐derived MV vortex ring parameters correlated strongly with the corresponding E and A wave peak velocities (see Table 3). Moreover, their correlations were stronger than the correlations to all systolic LV function parameters (Supporting Information Table S2).

For healthy subjects, early and late diastolic peak values of MV vortex ring parameters did not differ significantly, except for *CI* (*P* < .01). Correspondingly, *CI* was higher at E wave peak velocity frame than at A wave peak velocity frame (*CI*_E_ = 0.77 ± 0.07, *CI*_A_ = 0.66 ± 0.02, *P* < .01). Neither volume nor α, on the other hand, differed significantly between E wave peak velocity frame and A wave peak velocity frame (*vol*_E_ = 21 ± 4 mL, *vol*_A_ = 18 ± 3 mL, *P* = .12; α_E_ = 69º ± 12°, α_A_ = 71º ± 8°, *P* = .49).

## 4 DISCUSSION

We present an automated method for temporal MV vortex ring core and region extraction from 4D‐flow data allowing for quantitative vortex property assessment in healthy and diseased subjects. In particular, we demonstrate (1) substantial agreement with visual analysis, (2) robustness to manual LV segmentation speed‐up strategies, and (3) strong correlations between MV vortex ring parameters and transmitral peak velocities as well as differences in early diastolic MV vortex ring properties between healthy controls and patients with IHD.

### 4.1 Automated MV vortex ring extraction and analysis

As MV vortex rings were detected in both healthy controls and patients with severe IHD, the proposed algorithm can be considered a robust method for automated MV vortex ring extraction. The identification of MV vortex ring formation during early and late diastolic filling, with typical vortex dissolution during diastasis, is in accordance with visual observations by Elbaz et al.^13^ It has been further described previously that the MV vortex ring shape is not strictly toroid and changes over time, as the vortex entrains ventricular blood, is deformed, and interacts with ventricular structures, such as papillary muscles.^6^ By detecting different MV vortex ring shapes, the proposed algorithm is not only capable of extracting non‐toroid MV vortex rings, but also meets the requirements for analyzing the deforming MV vortex ring over time.

Automated calculation of MV vortex ring core and region parameters in each frame permits analysis of parameter‐time curves and their characteristics, such as the maxima or minima of parameters. While velocity‐derived MV vortex ring parameters resemble the biphasic time course of E and A wave velocities, no distinct patterns for temporal evolution of MV vortex ring volume, circularity, or angle to LV long axis were identified. This can probably be attributed to the fact that the evolving MV vortex ring adapts to intraventricular structures^12^ and may undergo vortex pinch‐off or merging processes.

### 4.2 Comparison with visual analysis

Automatically and visually detected MV vortex rings showed good agreement. The differences in MV vortex ring onset times can be explained by the difficulty of identifying subtle vortex structures visually. In frames at the end of the MV vortex ring evolution process, in which the MV vortex ring breaks and twists open, there were few cases where visual analysis detected an MV vortex ring but automated analysis did not, and the other way around. It is debatable whether the structures in these frames can still be considered MV vortex rings.

### 4.3 Dependence on segmentation

Automated MV vortex ring extraction was found to be robust when using accelerated manual LV segmentation approaches. According to the results of the slice distance experiment, segmentation time of 4D‐flow magnitude images can be reduced by segmenting fewer LV short‐axis slices. In particular, automated MV vortex ring extraction might be feasible with registered LV contours, such as from balanced SSFP cine short‐axis images (typical slice distance = 8‐10 mm), for which automated segmentation algorithms exist.^29^, ^30^ By applying a static end‐diastolic LV segmentation mask to all 30 cardiac frames, further substantial segmentation speed‐up can be achieved. Using mask_end‐dia_ resulted in acceptable MV vortex ring core and region extraction scores and yielded small MV vortex ring parameter errors. This finding is in line with the overall observation that MV vortex ring extraction is less sensitive to over‐segmentation than to under‐segmentation, which is to be expected, as in the latter case parts of the MV vortex ring core and region might be missed.

### 4.4 MV vortex ring parameters in healthy and diseased subjects

Apart from shape and position parameters,^6^, ^13^, ^31^ the literature lacks normal values describing the MV vortex ring in 3D. The observation that end‐diastolic MV vortex ring volumes in our healthy controls were smaller than the normal values given by Arvidsson et al^6^ can be explained by the conceptual difference between our Eulerian (based on the instantaneous velocity field) and their Lagrangian vortex detection method,^12^ which visualizes the history of flow and, therefore, mixes effects of early and late diastolic MV vortex rings. Furthermore, Lagrangian coherent structure analysis in the LV allowed only for identification of the leading edge, but not the trailing edge of the vortex.^12^

Automatically determined *CI* confirmed that in healthy subjects E vortex rings have a more circular shape than A vortex rings.^13^ Moreover, *CI* values at E and A wave peak velocity frames were within the previously published normal range.^32^ Interestingly, a difference in early diastolic (but not late diastolic) *CI* between healthy and diseased subjects, as reported by Calkoen et al^32^ for patients with corrected atrioventricular septal defect, was also found for the IHD patient cohort of the present study. The MV vortex ring core to LV long‐axis angle α did not differ significantly between E and A wave peak velocity frames, which is in accordance with previous publications.^13^, ^32^ Furthermore, angles to LV long axis were within the 95% confidence interval for healthy controls defined in a previous MV vortex ring shape study.^31^

Vorticity^33^, ^34^ and kinetic energy^5^, ^31^, ^34^, ^35^, ^36^, ^37^, ^38^, ^39^ from 4D‐flow data have mostly been quantified for the entire LV. Only one experimental study on pigs calculated the mean vorticity in the anterior and posterior 2D‐cut plane of the MV vortex ring.^11^ Average vorticity values around E and A wave peak velocity frames in that study (60‐80 s^−1^) are in a similar range as the 3D mean vorticity in our healthy controls. Kim et al^8^ measured absolute kinetic energy in a 2D slice of the MV vortex ring, which may explain why the early diastolic peak normal value was smaller than that in the present study. The larger early diastolic peak absolute kinetic energy observed by Kanski et al^5^ can again be explained by the difference between Eulerian and Lagrangian vortex detection criteria.

The observed differences in early diastolic MV vortex ring properties between healthy controls and patients with IHD supports the assumption that MV vortex ring alterations are associated with LV dysfunction. The strong correlations of velocity‐derived MV vortex ring parameters with their corresponding E and A wave peak velocities together with the weaker correlations with systolic LV function parameters suggest that they could serve as indices of LV diastolic function.^40^, ^41^ Moreover, differences of early and late diastolic peak values of MV vortex ring vorticity and kinetic energy between healthy controls and patients with IHD seem to resemble the different E‐to‐A‐wave peak velocity ratios between these groups. However, the precise relationship between MV vortex ring parameters and LV diastolic function remains to be evaluated in larger cohort studies.

### 4.5 Limitations

No reference standard exists for MV vortex ring detection. However, visual analysis is the most intuitive way of observing a vortex, and was therefore chosen as the reference method in this study.

4D‐flow data have limited spatial and temporal resolution, which might have contributed to the observation of U‐shaped and bracket‐shaped rather than toroid MV vortex rings. In particular, the spatial resolution of the 4D‐flow data of the present study is lower in the* z*‐direction than the minimum spatial resolution (3 × 3 × 3 mm^3^) recommended by the 4D‐flow MRI consensus statement.^7^ The impact of specific acquisition technique and sequence parameters on automated MV vortex ring extraction has not been investigated and is a topic for future research.

4D‐flow data in patients were assessed after contrast agent, whereas no contrast agent was applied in healthy controls. Even though they affect the SNR of the data, measured velocities and velocity fields should not be affected by contrast agent.^7^, ^42^, ^43^

Manual segmentation was performed with prototype software that is optimized for 4D‐flow data preprocessing and visualization rather than segmentation, which explains the long manual segmentation times per subject. Segmentation time might be substantially decreased with suitable 4D‐flow data segmentation software.

The number of healthy controls and patients with IHD was small, and only one patient group was included. However, the focus of this study was to provide a proof of concept of automated MV vortex ring extraction in healthy and diseased subjects and, as discussed previously, segmentation could be accelerated substantially to enable investigations in larger patient cohorts.

## 5 CONCLUSIONS

A method for automated extraction of the temporal evolution of MV vortex ring core and region from 4D‐flow data is introduced, demonstrating good agreement with visual analysis and robustness with respect to LV segmentation. Differences of quantitative MV vortex ring parameters between healthy and diseased subjects as well as strong correlations with peak inflow velocities indicate the potential of MV vortex ring properties to characterize LV function and dysfunction.

## CONFLICT OF INTEREST

Gert Reiter is employed by Siemens Healthcare Diagnostics GmbH.

## Supporting information


**TABLE S1** Systolic LV function parameters for healthy controls and IHD patients. *Note:* End‐diastolic volume (*EDV*), end‐systolic volume (*ESV*), ejection fraction (*EF*), stroke volume (*SV*) and cardiac output (*CO*) were derived from stacks of balanced SSFP (bSSFP) short‐axis cine series together with bSSFP two‐chamber and four‐chamber view cine series for mitral valve (MV) base plane modeling using dedicated software (*syngo.via*, Siemens Healthcare, Erlangen, Germany). The *P* value refers to unpaired *t*‐test. Abbreviation: ns, not statistically significant
**TABLE S2** Correlogram of systolic LV function parameters with early and late diastolic peak values of MV vortex ring parameters for all subjects (*N* = 20). *Note:* The Pearson correlation coefficient is specified with its 95% confidence interval. Abbreviation: ns, not statistically significant
