# Supplementary material for: Automated mitral valve vortex ring extraction from 4D‐flow MRI
Source: Magn Reson Med. 2020 Jun 18;84(6):3396–408. doi: 10.1002/mrm.28361 (PMC7540523; doi:10.1002/mrm.28361)
Supplement: Supplementary file 1 — TABLE S1 Systolic LV function parameters for healthy controls and IHD patients. Note: End‐diastolic volume (EDV), end‐systolic volume (ESV), ejection fraction (EF), stroke volume (SV) and cardiac output (CO) were derived from stacks of balanced SSFP (bSSFP) short‐axis cine series together with bSSFP two‐chamber and four‐chamber view cine series for mitral valve (MV) base plane modeling using dedicated software (syngo.via, Siemens Healthcare, Erlangen, Germany). The P value refers to unpaired t‐test. Abbreviation: ns, not statistically significant TABLE S2 Correlogram of systolic LV function parameters with early and late diastolic peak values of MV vortex ring parameters for all subjects (N = 20). Note: The Pearson correlation coefficient is specified with its 95% confidence interval. Abbreviation: ns, not statistically significant [file MRM-84-3396-s001.pdf]

Supporting Information

to **Automated mitral valve vortex ring extraction from 4D flow MRI**

Study population - details

Coronary artery disease was confirmed in all chronic ischemic heart disease (IHD) patients by coronary angiography; seven patients demonstrated subendocardial late Gadolinium enhancement compatible with the presence of chronic myocardial infarction scar. Whereas body surface area was larger (1.98 ± 0.17 m^2 vs. 1.76 ± 0.14 m^2, $P < 0.01$) and heart rate was smaller (60 ± 9 bpm vs. 69 ± 9 bpm, $P = 0.03$) in the chronic IHD patient group compared to the healthy controls, age (62 ± 8 years vs. 59 ± 7 years) and systolic left ventricular (LV) function parameters (Supporting Information Table S1) did not differ significantly.

Supporting Information Table S1: Systolic LV function parameters for healthy controls and IHD patients.

parameter	controls (*N* = 10)	IHD patients (*N* = 10)	*P* value
EDV (mL)	122 ± 23	137 ± 29	ns
ESV (mL)	47 ± 13	59 ± 22	ns
EF (%)	62 ± 6	58 ± 10	ns
SV (mL)	74 ± 13	78 ± 16	ns
CO (L/min)	5.2 ± 1.5	4.6 ± 0.9	ns

Note: End-diastolic volume (*EDV*), end-systolic volume (*ESV*), ejection fraction (*EF*), stroke volume (*SV*) and cardiac output (*CO*) were derived from stacks of balanced SSFP (bSSFP) short-axis cine series together with bSSFP two-chamber and four-chamber view cine series for mitral valve (MV) base plane modeling using dedicated software (*syngo.via*, Siemens Healthcare, Erlangen, Germany). The *P* value refers to unpaired *t*-test. Abbreviation: ns, not statistically significant.

Correlations of MV vortex ring parameters with systolic LV function parameters

Early and late diastolic peak values of MV vortex ring parameters demonstrated poor to moderate significant correlations with systolic LV function parameters (Supporting Information Table S2).

Supporting Information Table S2: Correlogram of systolic LV function parameters with early and late diastolic peak values of MV vortex ring parameters for all subjects (*N* = 20).

parameter	*EDV*	*ESV*	*EF*	*SV*	*CO*
**E vortex ring volume**	0.47 [0.03, 0.75]	0.60 [0.21, 0.82]	-0.50 [-0.77, -0.08]	ns	ns
**A vortex ring volume**	0.59 [0.20, 0.82]	0.60 [0.21, 0.82]	-0.48 [-0.76, -0.04]	ns	ns
**E vortex ring max vorticity**	ns	-0.45 [-0.74, -0.01]	0.61 [0.23, 0.83]	ns	0.45 [0.00, 0.74]
**A vortex ring max vorticity**	ns	ns	0.47 [0.04, 0.76]	0.45 [0.01, 0.75]	0.62 [0.25, 0.83]
**E vortex ring mean vorticity**	ns	ns	0.54 [0.13, 0.79]	ns	0.47 [0.04, 0.76]
**A vortex ring mean vorticity**	ns	ns	0.50 [0.08, 0.77]	0.51 [0.09, 0.78]	0.57 [0.17, 0.81]
**E vortex ring absolute E_{kin}**	ns	ns	ns	ns	ns
**A vortex ring absolute E_{kin}**	ns	ns	ns	0.50 [0.08, 0.77]	0.61 [0.24, 0.83]
**E vortex ring relative E_{kin}**	ns	ns	0.50 [0.07, 0.77]	ns	ns
**A vortex ring relative E_{kin}**	ns	ns	ns	0.53 [0.11, 0.79]	0.61 [0.23, 0.83]
**E vortex ring α**	ns	ns	0.47 [0.04, 0.76]	ns	0.45 [0.00, 0.74]
**A vortex ring α**	ns	ns	ns	ns	-0.52 [-0.78, -0.10]
**E vortex ring *CI***	ns	ns	ns	ns	ns
**A vortex ring *CI***	ns	ns	ns	ns	ns

Note: The Pearson correlation coefficient is specified with its 95% confidence interval. Abbreviation: ns, not statistically significant.
